# Novel and Alternative Targets Against Breast Cancer Stemness to Combat Chemoresistance

**DOI:** 10.3389/fonc.2019.01003

**Published:** 2019-10-16

**Authors:** Sangita Sridharan, Cory M. Howard, Augustus M. C. Tilley, Boopathi Subramaniyan, Amit K. Tiwari, Randall J. Ruch, Dayanidhi Raman

**Affiliations:** ^1^Department of Cancer Biology, University of Toledo, Toledo, OH, United States; ^2^Department of Pharmacology and Experimental Therapeutics, University of Toledo, Toledo, OH, United States

**Keywords:** breast cancer stemness, chemoresistance, therapy failure, CSC-directed therapy, novel targets, plasticity, minimal residual disease

## Abstract

Breast cancer stem cells (BCSCs) play a vital role in tumor progression and metastasis. They are heterogeneous and inherently radio- and chemoresistant. They have the ability to self-renew and differentiate into non-BCSCs. These determinants of BCSCs including the plasticity between the mesenchymal and epithelial phenotypes often leads to minimal residual disease (MRD), tumor relapse, and therapy failure. By studying the resistance mechanisms in BCSCs, a combinatorial therapy can be formulated to co-target BCSCs and bulk tumor cells. This review addresses breast cancer stemness and molecular underpinnings of how the cancer stemness can lead to pharmacological resistance. This might occur through rewiring of signaling pathways and modulated expression of various targets that support survival and self-renewal, clonogenicity, and multi-lineage differentiation into heterogeneous bulk tumor cells following chemotherapy. We explore emerging novel and alternative molecular targets against BC stemness and chemoresistance involving survival, drug efflux, metabolism, proliferation, cell migration, invasion, and metastasis. Strategic targeting of such vulnerabilities in BCSCs may overcome the chemoresistance and increase the longevity of the metastatic breast cancer patients.

## Introduction

Breast cancer (BC) is the second leading cause of death in women among the cancer mortalities. With the new estimates in 2019, 3 out of 10 women (30%) will develop BC in her lifetime and 1 in 7 (15%) will succumb to BC (1). Mortality in BC patients is mainly due to metastasis to the lungs, bone and the brain. Breast cancer is a heterogeneous disease with differential expression of several molecular markers. Luminal BC expresses estrogen receptor (ER), progesterone receptor (PR), and human epidermal growth factor receptor 2 (HER2). The triple-negative breast cancer (TNBC) subtype lacks the expression of all of the above three markers. Based on the gene expression profile, TNBC is classified into basal-like1 (BL1) and basal-like2 (BL2), mesenchymal (M), mesenchymal stem-like (MSL), immunomodulatory (IM), and luminal androgen receptor (LAR) types (2, 3). The pathological complete response (pCR) rate with BL1, BL2, LAR, and MSL tumors are 52, 0, 10, and 23%, respectively (4). Moreover, intra- and inter-tumor heterogeneity (clonal diversity), and plasticity observed in TNBC (5–11) leads to chemoresistance, tumor relapse, and poor patient outcome. Both luminal and TNBC are reported to contain a small subpopulation of cells amidst the bulk tumor cells called breast cancer stem cells (BCSCs) or tumor initiating cells. BCSCs are capable of self-renewal, tumor initiation and differentiation into bulk tumor cells. BCSCs are intrinsically chemoresistant and can repopulate the tumor following chemotherapy and ionizing radiation. This ultimately leads to therapy failure, distant metastasis, or metastasis of the metastases, tumor relapse and mortality. This is especially true in luminal HER2^+^ and TNBC tumor types. TNBC is highly lethal (5-year mortality >75%), characterized by aggressive growth, therapy failure, and lack of successful targeted therapies (12, 13). TNBC patients often exhibit initial sensitivity to neoadjuvant chemotherapy, but eventually become refractory to such therapy presumably due to BCSCs. BCSCs are thus clinically important and there is an unmet need to co-target BCSCs along with bulk tumor cells. The co-targeting approach may overcome chemoresistance, molecular and metabolic plasticity, and most importantly reduce mortality and improve longevity in metastatic BC patients (14–18). In order to design effective and rational therapies against BCSCs, it is imperative to find novel and actionable molecular targets to combine effectively with current available therapies. This review focuses on emerging molecular targets that could become potential BCSC-directed precision therapies to overcome clinical chemoresistance.

## Heterogeneity in BC and Its Influence on Clinical Outcome

The heterogeneity in BC cells can arise by stochastic genetic or epigenetic (clonal evolution) changes. The paracrine interactions of the tumor cells with its microenvironment (TME) can also confer phenotypic and functional heterogeneity based on the spatiotemporal dynamics (location of the tumor and continual changes in the cellular and acellular TME) of the constantly evolving tumors in the same patient (19–22). Metabolic heterogeneity has been reported to exist in BC organoids by employing optical metabolic imaging (OMI) similar to *in vivo* situation (23). OMI has been suggested to predict any potentially unresponsive subpopulation of cells within the tumor.

Heterogeneity exists among BCSCs as well (24). By isolating BCSCs based on high flavin content, energetic BCSCs (e-BCSCs) were identified with a higher glycolytic activity and a larger mitochondrial mass (25). On the contrary, quiescent BCSCs (qBCSCs) have been reported based on the epigenetic activities (26). Mesenchymal and epithelial phenotypes of heterogeneous BCSCs have been described contributing to differential chemoresistance (27). Notch-Jagged signaling has been proposed to contribute to heterogeneity in BCSCs with more mesenchymal BCSCs at the invasive edge and the hybrid epithelial/mesenchymal (E/M) BCSCs in the center of the tumor (24). Interestingly, ITGB4^+^-enriched BCSCs have been reported to reside in an intermediate E/M phenotypic state (28). Mathematical modeling coupled with data on single-cell sequencing of BCSCs has been suggested to dissect the heterogeneity. This will also help our understanding of the replication and invasive dynamics of BC cells during cancer progression and importantly in response to therapy (29).

Single cell sequencing (sc-seq) technology (single-cell genomics and transcriptomics) has pioneered our understanding of intra-tumoral genetic heterogeneity, the cancer genome evolution and also phenotypic diversity (30–32). Understanding molecular and genetic variations at the single cell level and as an ensemble in the tumor will provide mechanisms of chemoresistance. Chemoresistance and relapse can also occur in patients undergoing combination chemotherapy. In such cases, tapping the circulating tumor cells (CTCs) by liquid biopsy would enable assessment of the tumor cells for any molecular or genetic changes following chemotherapy. Many of the CTCs are BCSCs and one can examine for ratios of BCSCs to tumor cells (CD44 vs. CD24 and ALDH staining) before, during and after therapy. The isolated CTCs/BCSCs can be subjected to sc-seq for genomic, epigenomic, and transcriptomic analysis. Using this approach, continuously activated T-cells were identified in the cellular TME. Additionally, it revealed a co-existence of M1 and M2 macrophage polarization genes in the same cell indicating that macrophages fall along a spectrum between the two states (33). Also, aldehyde dehydrogenase (ALDH^+^) positive BCSCs at the single cell level analysis, exhibited hybrid epithelial/mesenchymal phenotype with a gene expression associated with aggressive TNBC (34). Identification of biomarkers predictive of therapy response and emergence of resistance following therapy based on sc-seq would prove valuable (17).

### tRNA as Predictive Biomarkers in BCSCs

Transfer RNA (tRNA)-derived small non-coding RNAs (tDRs) are novel small non-coding RNAs (sncRNA) that have been demonstrated in some human diseases and biological processes. BCSCs isolated by the expression of CD44^+^/CD24^−/low^ surface markers were tested for tDR expression profiles in TNBC and non-TNBC types by RNA sequencing (RNA-Seq). Among a total of 1,327 differentially expressed tDRs, 18 were upregulated and 54 were downregulated in the TNBC group. The expression level of tDR-000620 was consistently lower in BCSCs derived from TNBC cell lines and patient serum samples. Interestingly, tDR-000620 expression (*p* = 0.002) and the node status (*p* = 0.001) groups were statistically significant with recurrence-free survival (35).

tRNA-derived fragments (tRF) also serve as predictive biomarkers (36). tRF-30-JZOYJE22RR33 and tRF-27-ZDXPHO53KSN were correlated with trastuzumab resistance (37). The tDRs such as tDR-0009 [derived from transfer RNA (tRNA)^Gly−GCC−1−1^] and tDR-7336 (derived from tRNA ^Gly−GCC−1−2^) were significantly upregulated when the SUM-1315 cell line was subjected to hypoxic conditions. The protein-protein interaction network from the STRING database identified that tDR-0009 may be involved in imparting chemoresistance to TNBC cells through the regulation of STAT3 activation. Specific tDRs act as regulatory factors in hypoxia-induced chemoresistance in TNBC, and they could serve as predictive biomarkers (38). In HER2-overexpressing breast cancer, there is an ongoing clinical trial evaluating molecular biomarkers to predict the efficacy of the Trastuzumab therapy and recurrence (NCT03521245).

## Breast Cancer Stem Cells

BCSCs through their self-renewal capacity can initiate tumorigenesis, contribute to primary tumor progression, local invasion, and distant metastases (39). Historically, CSCs have been described as a “side population” (SP) by flow cytometric analyses based on the exclusion of the Hoechst dye by the drug transporters in CSCs. This reflects their capability to exclude xenobiotics including anti-cancer drugs to outside of the cell. There is spatial and temporal variability in the expression of stemness markers by BCSCs such as CD44 (Hyaluronan receptor) (39), CD133 (40, 41), CD49f^+^ (Integrin-α_6_) (42), epithelial cell adhesion molecule (EpCAM), chemokine receptor CXCR4, transcription factors [SRY (sex determining region Y) box 2—SOX2, homeobox protein Nanog, and octamer-binding transcription factor 4 (OCT4)] and aldehyde dehydrogenase (ALDH) activity (39). A small fraction of BCSCs express both CD44 and ALDH markers and are considered highly metastatic (39, 43). Interestingly, there are 2 isoforms of CD44 with opposite functions. The standard isoform of CD44 (CD44s) promotes BCSC stemness whereas the CD44 variant form (CD44v) opposes it (44). SOX2 works in conjunction with cyclin-dependent kinases 4/6 to transactivate the Cyclin D1 promoter, which facilitates proliferation and clonogenicity (45, 46). In TNBC, SOX2 promotes proliferation, and metastasis (47). SOX2 also promotes tamoxifen resistance (48) and a SOX2-SOX9 signaling axis was reported to maintain BCSCs (49). Resistance to tamoxifen by ER^+^-BCSCs was attributed to SOX9-FXYD Domain Containing Ion Transport Regulator 3 (FXYD3)-*Src* axis (50). Basically, significantly upregulated expression of FXYD3 is crucial for mediating tamoxifen resistance in ER^+^-BCSCs. FXYD3 is critical for the nuclear localization of SOX9 which in turn directly promotes the expression of FXYD3 forming a positive feedback loop. The trimeric complex consisting of FXYD3, ER-α and c-*Src* which transduces non-genomic estrogen signaling which facilitates the activity of ER^+^-BCSCs. Nanog is also involved in the maintenance of pluripotency and self-renewal of BCSCs. An increased expression of Nanog serves as a prognostic indicator and was suggested to be co-expressed with the CD133 marker (22, 51–53). OCT4 expression has been suggested to be a worse prognostic marker for surgical TNBC patients (54). Expression of SOX2, Nanog and OCT4 transcription factors correlated with poor differentiation, advanced BC stage and worst survival in BC patients with HER2 positivity (55). The expression of cell surface and subcellular markers of BCSCs is not a static property as they change in response to their microenvironment. Mesenchymal and epithelial phenotypes of BCSCs have been described with distinct gene expression profiles and contribute to heterogeneity and differential chemoresistance (27). The differential characteristics between these cells are described in Table 1. A hybrid version of BCSCs has been suggested to exist with both epithelial and mesenchymal stem cells markers in the center of the tumor (24, 28). Generally, mesenchymal BCSCs are more resistant to chemotherapy than the epithelial type (69). Interconversion between them occurs at a slow rate which we call “stem cell buffering” (SCB) (27, 70). The innate plasticity of BCSCs, thus contributes to tumor heterogeneity and chemoresistance. The BCSCs can dynamically oscillate between a bulk tumor cell type and stemness state based on temporal and spatial context in the microenvironment around the BCSC (22). For example, chemotherapy may first induce a BCSC phenotype conversion from bulk tumor cells. Following cessation of therapy, cells may revert to bulk tumor cells. Additionally, there is heterogeneity in BCSC pools in which the subsets of BCSCs have differing abilities ranging from quiescence, chemoresistance, interconversion between epithelial to mesenchymal types, proliferation, local invasion and metastasis. Thus, there is remarkable genetic and/or epigenetic heterogeneity and cellular plasticity in BCSCs and bulk tumor cells presenting clinical challenges. Thus, it is imperative to develop targeted therapies against the “mosaic nature” of BCSCs along with the co-targeting of bulk tumor cells.

**Table 1:** Differential characteristics of Mesenchymal vs. Epithelial BCSCs.

**Attribute**	**Mesenchymal BCSCs**	**Epithelial BCSCs**
Primary identifying markers	CD44^High^/CD24^Low^ (39)	ALDH activity and Western blotting for ALDH isozymes (56)
Location in the tumor	Tumor-invasive front in normoxic regions closer to the stroma (27)	Centrally located in the tumor within the internal hypoxic zones (27, 57).
Secondary identifying markers	EpCAM^−^, CD49f^+^, ESA^+^ (58)	EpCAM^+^, CD49f^+^ (58)
Breast cancer subtypes	Preponderance in basal and claudin-low, HER2-breast cancer subtypes (59–61)	High tendency to be found in HER2^+^, luminal breast cancers (62, 63)
Invasive and metastatic potential	Enhanced tendency to invade and metastasize, demonstrated by increased expression of proinvasive genes [IL-1α, IL-6, IL-8, CXCR4, MMP-1, and urokinase plasminogen activator (UPA)] (64)	ALDH^**+**^ cells are more aggressive in behavior and may predict metastasis (65, 66)
Chemokine receptor expression	–	Higher expression of chemokine receptors CXCR1 and CXCR2 (66)
Proliferation rate	Relatively quiescent as determined by the low expression of Ki 67 (56)	Ki67 is preferentially expressed in ALDH^+^ BCSCs making them relatively more proliferative (56)
Epithelial and mesenchymal traits	SAGE studies have shown higher levels of EMT-associated mRNA in CD44^**+**^/CD24^**−**^ BCSCs (67)	Associated with epithelial-like characteristics and gene expression (68)

*ALDH1, Aldehyde dehydrogenase 1; EpCAM, Epithelial cell adhesion molecule; CD49f^+^, α6-integrin; ESA, Epithelial specific antigen; HER2, Human epidermal growth factor receptor 2; MMP, Matrix metalloproteinase; SAGE, Serial analysis of gene expression; PARP1, Poly(ADP-Ribose) Polymerase 1; CXCR1, CXC-motif receptor 1; CXCR2, CXC-motif receptor 2*.

## The Link Between Epithelial-Mesenchymal Transition (EMT) and BCSCs

The mechanistic evidence suggests that EMT and the acquisition of BC stemness are correlated (69). Following the experimental activation of the EMT program (HMLER cells), induction of the autocrine signaling loops that were known to associate with cancer stemness were observed. Importantly, blocking the autocrine pathways was sufficient to abolish the CSC properties. This brings out the causal link between EMT and induction of BC stemness. The EMT program can also contribute to cancer stemness through its effects on intracellular signaling pathways. For instance, EMT-transcription factor (EMT-TF) Snail1 has been reported to diminish the expression of p53 in tumor cells through the formation of a ternary complex consisting of a Snail1, histone deacetylase 1 (HDAC1) and p53. This ternary association leads to deacetylation of p53 and its degradation (71). TGF-β signaling pathway has also been demonstrated to induce the expression of EMT-TFs such as Twist, Snail1, and Slug (72). In early breast cancer patients, a spectrum of EMT phenotypes in circulating tumor cells (CTCs) has been reported (73).

## The BC Stemness State Imparts Therapy Resistance in the Clinics

BCSCs possess the intrinsic ability to survive cytotoxic therapy through a variety of mechanisms. They include upregulation of anti-apoptotic proteins, activation of alternate survival pathways, drug efflux or ATP-binding cassette (ABC) transporters, detoxification/reduction of reactive oxygen species (ROS) (18, 74), and an enhanced capacity for DNA repair (75, 76). Myeloid cell leukemia 1 (MCL1) is one of the key proteins involved in the survival of BCSCs (77, 78). Both MYC and the anti-apoptotic protein MCL1 co-operate in BCSCs to promote chemoresistance through mitochondrial oxidative phosphorylation (79). Treatment of TNBC with “mammalian target of rapamycin complex 1/2” (mTORC1/2) inhibitors led to sustained drug-resistance in Notch1-dependent BCSCs (80). Interestingly, the Notch-mediated tumor-stroma-inflammatory network promoted tumor invasiveness and secretion of the chemokine CXCL8. CXCL8 promotes BC stemness through its action on the chemokine receptors CXCR1 and CXCR2 on BCSCs (81). The survival and resistance through upregulation and rewiring of alternate pathways in breast cancer is provided in Table 2.

**Table 2:** Resistance mechanisms encountered in BCSCs during or after therapy.

**FDA-approved breast cancer drugs**	**Drug target/mode of action**	**BCSC resistance mechanism**	**References**
Exemestane (Aromasin)	Small molecule inhibitor of aromatase	Exemestane induces AREG in an ER-dependent manner. AREG then activates the EGFR and downstream MAPK pathway, driving cell proliferation	(82)
Anastrozole	Small molecule inhibitor of aromatase	Causes resistance by constitutive activation of the PI3K/AKT/mTOR pathway	(83)
Letrozole (Femara)	Non-steroidal aromatase inhibitor	Treatment caused resistance by upregulation of HIF1-α target genes such as BCRP through activation of the PI3K/AKT/mTOR pathway	(84)
Cyclophosphamide (Clafen)	Crosslinks DNA and targets NR1/2	ALDH1A1 detoxifies the active form of Cyclophosphamide to an inactive metabolite. Treatment causes an NF-κB–IL-6–dependent inflammatory environment that induces stemness. Loss of PPARγ causes expansion of the CSC population resistant to cyclophosphamide. Mortalin (mtHsp70) upregulation leads to an increase in stem cell markers such as OCT4 and ALDH1 leading to drug resistance. Treatment-induced senescence greatly enhanced tumor stemness and relapse potential upon exit from the senescence state through the *Wnt* pathway	(85–87)
Doxorubicin hydrochloride, epirubicin	Cytotoxic anthracycline which intercalates with DNA and inhibits DNA topoisomerase	Doxorubicin-resistant cells had downregulated BRCA1/2, p53, Bcl2, and E-cadherin while upregulating glutathione-S-transferaseπ, PKCα, and ABC transporters. Treatment causes an NF-κB–IL6–dependent inflammatory environment that induces cancer stemness. Treatment caused an increase in the population of ALDH1^**+**^ tumor cells. Mortalin (mtHsp70) upregulation leads to increase in stem cell markers such as OCT4 and ALDH1 leading to drug resistance. CSCs resist treatment and confer resistance to nearby cancer cells through upregulation and exosome mediated secretion of miR155. Pygo2 upregulation (Wnt/β-catenin pathway component) expanded the treatment-resistant stem cell population. CSCs repress expression of KRT19 leading to loss of nuclear import of the β-catenin/RAC1 complex causing downregulation of NUMB and upregulation of NOTCH, ultimately imparting drug resistance to the CSCs. Overexpression of the microRNA106b~25 cluster conveys resistance by repressing EP300 (a transcriptional activator of E-cadherin) leading to a more EMT phenotype and an increase in stemness. Loss of CRB3 led to TAZ overexpression which enhanced metastatic capability and drug resistance in CSCs. ECM1 overexpression caused an increase in β-catenin expression enhancing the stem cell phenotype and associated drug resistance. TLR3 activation induces the β-catenin pathway which promotes CSC drug resistance. A feedback loop between AURKA and FOXM1 are crucial for stem cell self-renewal and are upregulated in drug resistant cell lines. p62 delays MYC mRNA degradation by repressing let7a and let7b enhancing stemness in resistant cell lines. The miR200b-Suz12-cadherin pathway promotes CSC growth and drug resistance. RNF8 activates Twist via ubiquitination and causes its localization to the nucleus where it promotes EMT and the CSC phenotype leading to drug resistance. Treatment eliminates less aggressive CSCs leaving behind an aggressive PDGFR signaling-driven CSC population that has PKCα-dependent activation of FRA1 which drives EMT	(87–100)
5-Fluorouracil	Targets uridine phosphorylase and SOD1	Selectively induced expression of the ADAM12L isoform leading to increased expression of pAKT levels. Treatment causes an NF-κB–IL6–dependent inflammatory environment that induces cancer stemness. Treatment increased the number of CSCs and their self-renewing capability in cells with high expression of CDK4	(85, 101, 102)
Gemcitabine Hydrochloride	Targets DNA by replacing cytidine causing arrest in DNA replication	Resistance correlated with increased activity of the PI3K/AKT pathway	(103)
Fulvestrant (Faslodex)	Estrogen receptor antagonist	Led to increased stem cell activity through activation of the JAG1-NOTCH4 receptor pathway. Treatment caused upregulation of SOX2 and Wnt pathways. Therapy led to reduced ERα expression but also increased IL-6 expression which drove stemness and resistance in CD133 high cells	(40, 104)
Docetaxel, Paclitaxel	Taxane, antimitotic chemotherapeutic that primarily targets microtubules and their associated proteins	Treatment creates an environment that allows for expansion of a CD49f^**+**^ chemoresistant population with tumor initiating capability. Treatment caused an increase in the population of ALDH1^**+**^ tumor cells. Resistance was found to be mediated by downregulation of miR27b leading to an increase in the level of ENPP1 which promotes expression of ABCG2. Treatment increased the number of CSCs and their self-renewing capability in cells with high expression of CDK4. LSD1-mediated resistance by upregulation of EMT related genes and pathways such as the PI3K/AKT pathway, leading to active induction of the CSC phenotype. Loss of SOCS3 leads to increased IL-6 mediated NF-κB signaling, increasing the BCSCs in p53/PTEN breast cancer cells. CSCs resist treatment and confer resistance to nearby cancer cells through upregulation and exosome mediated secretion of miR155. TAZ overexpression leads to enhanced metastatic capability and drug resistance in CSCs. TLR3 activation induces the β-catenin pathway which promotes CSC drug resistance. IRAK1 is phosphorylated upon treatment (paclitaxel) inducing inflammatory cytokine expression and enrichment of drug-resistant CSCs. A feedback loop between AURKA and FOXM1 are crucial for stem cell self-renewal and are upregulated in drug resistant cell lines. An axis of SOX2, ABCG2 and TWIST1 promotes pluripotency and resistance in CSCs. Treatment induced BDNF which promoted self-renewal and drug resistance of TrkB^**+**^ CSCs through KLF4. Treatment activates glucocorticoid receptors leading to an increase in YAP which causes an increase in drug-resistant CSCs. A drug-resistant CD10^**+**^, GPR77^**+**^- CAF population secretes IL-6 and IL-8 promoting stemness and drug resistance in cancer stem cells. CSCs and their drug resistance depend on HN1L to sustain activation of the LEPR-STAT3 pathway. Treatment eliminates less aggressive CSCs leaving behind an aggressive PDGFR signaling-driven CSC population that has PKCα dependent activation of FRA1 which drives EMT. The JAK/STAT3 pathway is upregulated in drug-resistant CSCs through CPT1B expression and fatty acid β-oxidation activity	(42, 90, 91, 93, 94, 99–101, 105–115)
Paclitaxel + Dasatinib combination therapy	Paclitaxel: See above Dasatinib: Inhibitor of the BcrAbl and Src kinase family	Paclitaxel treatment induced Dasatinib resistance by increased activation of several molecules involved in survival, malignancy, or stemness such as OCT3/4, Nanog, SOX2, c-*MYC*, c-*Src*, and Notch 1	(116)
Trastuzumab (Herceptin)	A neutralizing antibody against the extracellular domain of the EGFR protein	3D architecture results in enhanced BCSC population and modulates HER2 distribution, leading to increased Trastuzumab resistance. Treatment increased the frequency of EMT-like cancer CSCs in HER2^+^, PTEN^−^ cells through an IL-6 inflammatory feedback loop. miR-2055p is overexpressed in cancer, directly represses HER2, and indirectly represses EGFR through p63 leading to resistance of targeted therapy	(117–120)
Tamoxifen Citrate	Selective estrogen receptor modulator, acting as an inhibitor in mammary tissue	Tamoxifen treatment was found to induce pluripotency related phenotype in ERα-positive breast cancer cells. This was associated with relapse of tumors expressing enhanced levels of ALDH1A1	(121)
Radiotherapy	Induces DNA damage	ATM phosphorylates and stabilizes ZEB1 which then interacts with USP7 to stabilize CHK1, promoting resistance to radiotherapy in CSCs	(122)
Sirolimus, Everolimus	mTOR inhibitors	The reprogramming of cells upregulates EVI1 and SOX9, causing an increased expression of key mTOR pathway components such as RAPTOR, ultimately increasing the stem-like signature	(123)
Methotrexate	Inhibitor of tetrahydrofolate dehydrodgenase	Mortalin (mtHsp70) upregulation leads to an increase in stem cell markers such as OCT4 and ALDH1 leading to drug resistance	(87)
Lapatinib	Small molecule inhibitor of HER2 and EGFR	miR-2055p is overexpressed in cancer, directly represses HER2, and indirectly represses EGFR through p63 leading to therapy resistance. Integrin α_v_β_3_ drives the KRAS–RaIB–NF-κB pathway leading to enhanced stemness and resistance	(119, 124)

*CSCs, Cancer stem cells; EGFR, Epidermal growth factor receptor; AREG, Amphiregulin; ER, Estrogen receptor; MAPK, Mitogen activated protein kinase, PI3K, Phosphatidylinositol-3-kinase, AKT (PKB), protein kinase B; mTOR, Mechanistic target of rapamycin; HIF1, Hypoxia inducible factor1; BCRP (ABCG2), Breast cancer resistance protein (ATP binding cassette subfamily G member 2); ALDH1, Aldehyde dehydrogenase 1; NFκB, nuclear factor kappa light chain enhancer of activated B cells; IL-6, Interleukin-6; BRCA1/2, Breast cancer gene 1 and 2; Bcl2, Bcell lymphoma2; PKC, Protein kinase C; ABC, ATP Binding cassette transporters; ADAM12, Disintegrin and metalloproteinase domain containing protein 12; JAG1, Jagged 1; NOTCH4, Neurogenic locus notch homolog protein 4; SOX2, SRY (sex determining region Y) box 2; Oct3/4, Octamer binding transcription factor 3/4; HER2, human epidermal growth factor receptor 2; ZEB1, Zinc Finger E-Box Binding Homeobox 1; USP7, Ubiquitin Specific Peptidase 7; CHK1, Checkpoint kinase 1; EVI1, Ecotropic virus integration site 1; SOX9, SRY (sex determining region Y) box 9; RAPTOR- Regulatory associated protein of mTOR; Hsp70, Heat shock protein family A member 2; miR, microRNA; ENPP1, ecto nucleotide pyrophosphatase/phosphodiesterase family member 1; EMT, Epithelial to mesenchymal transition; PTEN, Phosphatase and tensin homolog; SOCS3, Suppressor of cytokine signaling 3; KRT19, Keratin19; RAC1, Ras-related C3 botulinum toxin substrate 1; NUMB, Protein numb homolog; TAZ, transcriptional coactivator with PDZ binding motif; CRB3, Crumbs protein homolog 3; ECM1, Extracellular Matrix Protein 1; TLR3, Toll-like receptor 3; IRAK1, interleukin1 receptor associated kinase 1; AURKA, Aurora kinase A; FOXM1, Forkhead box subclass M1; KRAS, Kras; RalB- RAS-like protooncogene B; TWIST1, Twist Family BHLH Transcription Factor 1; BDNF, Brain derived neurotrophic factor; TrkB, Neurotrophic Receptor Tyrosine Kinase 2; KLF4, Kruppel like factor 4; YAP, Yes-associated protein; IL-8, Interleukin-8; PDGFR, Platelet derived growth factor receptor; FRA1, Fos-like 1, AP1 Transcription Factor Subunit; HN1L, Hematological and neurological expressed 1like protein; LEPR, Leptin Receptor; STAT, Signal transducer and activator of transcription; JAK, Janus Kinase; CPT1B, Carnitine Palmitoyltransferase 1B*.

## Resistance Arising From BCSCs and the Tumor Microenvironment

**Figure 1:**
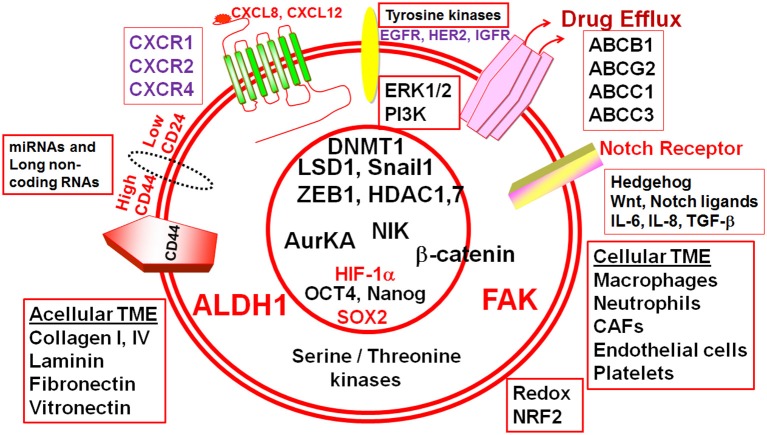
Cellular and acellular tumor microenvironment (TME) shape the response of breast cancer stem cells (BCSCs). The schematic diagram depicts different molecular players that execute the functionality of BCSCs and form potential actionable molecular targets in BCSCs. ALDH1, Aldehyde dehydrogenase 1; CD44, Cluster differentiation antigen 44; CD24, Cluster differentiation antigen 24; EGFR, Epidermal growth factor receptor; HER2, Human epidermal growth factor receptor 2; IGFR, Insulin-like growth factor receptor; CXCR1, CXC-motif receptor 1; CXCR2, CXC-motif receptor 2; CXCR4, CXC-motif receptor 4; ERK1/2, Extracellular signal regulated kinase1/2; PI3K, Phosphatidylinositol-3-kinase, FAK, Focal adhesion kinase; HIF-1α, Hypoxia inducible factor-1α; ABC, ATP Binding cassette transporters; BCRP (ABCG2), Breast cancer resistance protein (ATP binding cassette subfamily G member 2); ABCB1, ATP binding cassette subfamily B member 1; ABCC, ATP binding cassette subfamily C member 1; ABCC3, ATP binding cassette subfamily C member 3; IL-6, Interleukin-6; IL-8, Interleukin-8; SOX2, SRY (sex determining region Y) box 2; OCT4, Octamer binding transcription factor 4; ZEB1, Zinc Finger E-Box Binding Homeobox 1; miR, microRNA; AURKA, Aurora kinase A; NIK, NF-kB inducible kinase; AurKA, Aurora Kinase A; LSD1, Lysine-specific demethylase1; HDAC1, Histone deacetylase1; HDAC7, Histone deacetylase7; DNMT1, DNA methyltransferase1; TGF-β, Transforming growth factor-β; NRF2, NF-E2-related factor 2.

**Figure 2:**
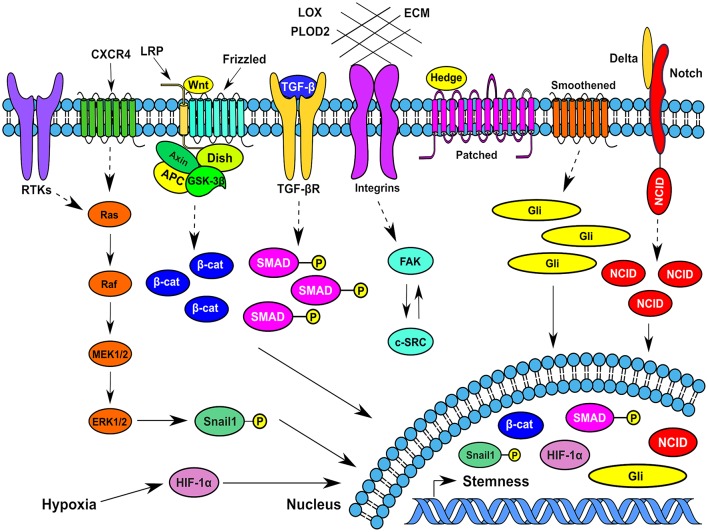
Typical signaling pathways operating in breast cancer stem cells at different spatiotemporal contexts in the tumor microenvionment. Several signaling pathways that function in BCSCs impinge on transcription factors such as Snail1, β-catenin, Gli1, HIF-1α, phospho-SMADs, and Notch intracellular domain that traverses to the nucleus and increase or maintain the breast cancer stemness. CXCR4, CXC-motif receptor 4; RTKs, Receptor tyrosine kinases; MEK1/2, Mitogen activated protein kinase kinase 1/2; ERK1/2, Extracellular signal regulated kinase1/2; HIF-1α, Hypoxia inducible factor-1α; β-cat, β-catenin; ECM, Extracellular Matrix; FAK, Focal adhesion kinase; Gli- a transcription factor; SMAD, Homologous to Caenorhabditis elegans SMA (“small” worm phenotype) and Drosophila MAD (“Mothers Against Decapentaplegic”); Dish, Disheveled; Hedge, Hedgehog; APC-Adenomatous Polyposis Coli; c-SRC, cellular protooncogene similar to viral sarcoma; GSK-3β, Glycogen synthase kinase-3β; LPR, Lipoprotein receptor related protein; TGF-β, Transforming growth factor-β; NCID, Notch intracellular domain; LOX, Lysyl oxidase; PLOD2, Procollagen-lysine,2-oxoglutarate 5-dioxygenase.

Currently, there are no clearly defined, targeted inhibitors for BCSCs established for successful BCSC-directed therapy. One of the key considerations for such targeted therapy is that the selected targets should be enriched in BCSCs. If the target is not enriched, at least the relative susceptibility of BCSCs should be present. Alternatively, dual targeting of BCSCs and bulk tumor cells with synergistic inhibitors may prevent activation of alternative survival pathways and subsequent chemoresistance. Generally, one should avoid targeting molecular nodes that are common to both BCSCs and normal mammary stem cells (MaSCs) or other stem cells in the body. At the least, the developed inhibitors should be minimally toxic to MaSCs. Alternatively, one has to specifically deliver drugs that have efficacy against BCSCs by employing a targeted delivery approach such as nanotechnology (tumor-homing nanoparticles or nanospheres) (125). In addition to paracrine input from bulk tumor cells, BCSCs depend on the surrounding tumor microenvironment (TME) called the “BCSC niche.” The “BCSC niche” is currently a high value therapeutic target. BCSCs interact constantly with the cellular component of the niche including neutrophils, macrophages, endothelial, and endothelial progenitor cells, mesenchymal stem cells and carcinoma associated fibroblasts (CAFs) (126–128). The signaling cues from the acellular TME such as cytokines, chemokines, growth factors, and some hormones activate many signaling pathways in BCSCs and form attractive targets in BCSCs (Figures 1, 2). For example, the chemokine CXCL8 or interleukin-8 (IL-8) and the hormone erythropoietin activated survival signaling pathways protect BCSCs following chemotherapy (129–131). Inflammatory components from TME also feed into BCSCs. Inhibition of cyclooxygenase-2 (COX-2) led to blockade of TGFβ-induced enrichment of two morphologically distinct BCSC populations; CD44^hi^/CD24^lo^ and ALDH^+^ (43, 132). The role of hedgehog (Hh), Notch, and Wnt signaling pathways in CSCs has been reviewed previously (53). Additionally, efflux transporters are also implicated in clonogenicity, pluripotency, and survival of BCSCs against cytotoxic chemotherapy (133, 134).

## Emerging Targets for Breast Cancer Stem Cells

### Efflux Transporters

The intrinsic multidrug resistance (MDR) in BCSCs determines the efficacy of chemotherapy. One of the key characteristics that differentiate BCSCs from normal cells is an increased expression of ATP-binding cassette (ABC) efflux transporters. Upregulated expression of ABC transporters may contribute tremendously to chemoresistance. It is imperative to target these transporters in BCSCs without running into toxicity problems. Of the 49 ABC transporters (ABCT) known, ABCB1 [P-glycoprotein (Pgp) or multidrug resistance protein 1 (MDR1)], ABCC1 [(multidrug resistance-associated protein 1 (MRP1)], ABCC3, and ABCG2 [(breast cancer resistance protein (BCRP)] protect BCSCs from drugs by exporting them out of the cells. Among these, ABCG2 and ABCB1 serve as functional cell surface markers for BCSCs. Preclinical studies indicated that genetic deletion of ABCG2 significantly reduced the number of normal SP cells but nearly ablated them in mammary glands of Abcb1a/1b^−/−^; Abcg2^−/−^ mice. Also, knockdown of ABCC3 led to a reduction in stemness. Additionally, there was a reduction in the number of BCSCs bearing CD44 on their cell surface. Importantly, the knockdown of ABCC3 demonstrated reduced formation of primary tumors (tumor initiating ability) and more susceptibility to doxorubicin in a xenograft mouse model (135). Hypoxic regions observed in a rapidly growing tumor can induce the expression of the transcription factor hypoxia inducible factor1-α (HIF1-α). HIF1-α can in turn induce the expression of ABCB1 which results in an expansion of the BCSCs via paracrine activation by interleukin-6 (IL-6) and CXCL8 (136). It is reported that ABC transporters may increase chemoresistance through expansion of BCSCs (75, 120). So, targeting of MDR is highly important because even if the BCSC targets or signaling nodes are highly sensitive, the effective drugs may simply be exported out. So achieving effective intracellular therapeutic concentration in BCSCs is crucial in eliminating them.

Several approaches have been attempted to overcome MDR including (i) ABC gene silencing by anti-sense oligonucleotide (ASO)inhibitors (ii) Inhibition of the functionality of ABCT through competitive and allosteric modulators (iii) miRNA-mediated downregulation (iv) Targeted inhibition of receptor tyrosine kinases (v) Nanoparticle-mediated delivery of inhibitors (vi) Transcriptional and post-translational regulation of ABCTs and (vii) Signaling pathways affecting them (76, 137). A few examples of the aforementioned approaches that were successful are provided here. Specifically, for instance, “third generation” ABCB1 modulators, such as elacridar (GF120918), laniquidar (R101933), zosuquidar (LY335979), and tariquidar (XR9576) with only nanomolar concentrations of the inhibitors required to inhibit ABCB1-mediated pumping of drugs out of tumor cells effectively. Elacridar was also found to inhibit ABCG2 transporter (138, 139). Therefore, this compound may be useful in treating MDR tumors that express multiple ABC transporters, specifically targeting CSCs (139). Tyrosine-kinase inhibitors (TKIs) such as erlotinib, lapatinib, imatinib, and nilotinib at clinically achievable concentrations, modulate the ATPase activity of ABC transporters, inhibiting the active drug export (140). Specifically, suppression of ABCB1 and/or ABCG2 by TKIs has been demonstrated in several studies, though the detailed mechanism remains unclear (141–144). The caveat is that overcoming MDR mediated by ABC transporters may prove difficult as selective targeting of the BCSCs is vital to avoid any detrimental side effects on normal breast, hematopoietic stem cell populations and the central nervous system. One potential approach would be to define a “therapeutic window” that selectively eliminates BCSCs without affecting normal stem cells. Another challenge is the observed compensatory mechanisms between ABC transporters. For example, several ABCTs have overlapping substrates, i.e., redundant substrate recognition which can lead to cross-resistance to specific drugs/xenobiotics. Such compensatory changes by ABCTs are problematic and will accentuate the tumor growth and chemoresistance. Understanding how functional redundancy among ABCTs contribute to self-renewal of BCSCs is immensely important (145). Finally, drugs targeting ABCTs (e.g., Lapatinib) may be delivered to BCSCs through nanocarriers and using antibodies like Trastuzumab directed against HER2. Alternatively, a combinatorial therapy involving low dose inhibition of ABCTs and another key target such as DNA repair capacity, ALDH/CD44 activity, reactive oxygen species (ROS), anti-apoptotic signaling nodes, key proteins that modulate autophagy/senescence, epigenetic modulators, modulators of EMT, and metastasis or radiotherapy may prove beneficial.

### G-Protein Coupled Receptors (GPCRs)

#### CXCR1 and CXCR2

Chemokine receptors CXCR1 and CXCR2 are Gα_i_-coupled receptors that generally play a role in chemotaxis of neutrophils, macrophages, and endothelial cells in a physiological microenvironment. In the breast cancer setting, they play a vital role in survival of BCSCs before and after chemotherapy (129–131). CXCL8 can activate both CXCR1 and CXCR2. These receptors not only feed into their own cell survival pathways, but also transactivate epidermal growth factor receptor (EGFR) and human epidermal growth factor receptor 2 (HER2) in BCSCs (146). Selective targeting of BCSCs with CXCR1 inhibitors would also facilitate reduction in pro-tumor stromal cells that express CXCR1 (147, 148). Antagonizing CXCR1 either by CXCR1-neutralizing antibody or by the small molecule inhibitor Repertaxin selectively depleted BCSCs than bulk tumor cells *in vitro*. This was followed by massive apoptosis of bulk tumor cells through FASL/FAS signaling through FAK/AKT/FOXO3A pathway. Furthermore, in a xenograft model, Repertaxin reduced the primary tumor burden and metastasis. It was proposed that CXCR1 blockade may selectively eliminate BCSCs (147). The chemotherapeutic drug Reparixin is an allosteric inhibitor of CXCR1 and CXCR2. A phase Ib trial (NCT02001974) has been conducted to evaluate the efficacy of reparixin in inhibiting CXCR1 and CXCR2 and any attendant toxicity (149). In this trial, co-administration of paclitaxel and reparixin appeared to be safe and tolerable in metastatic breast cancer. Additionally, the trial had demonstrated responses in the enrolled population. Based on the favorable outcome, this was taken for further study in a randomized Phase II trial and the results are awaited (NCT02370238).

#### CXCR4

The chemokine receptor CXCR4 is also a Gα_i_-coupled receptor. Physiologically, this chemokine receptor generally plays a role in embryogenesis. CXCR7, another Gα_i_-coupled receptor, shapes the CXCL12 (ligand) gradient for embryonic cells expressing CXCR4. As a result, these cells migrate and form different regions of the embryo. This system is recapitulated by the tumor cells. The chemokine receptor CXCR4 is expressed in BCSCs and is a key chemokine receptor involved in metastasis of breast cancer (150, 151) and forms a target in restraining or removal of BCSCs. Activation of this receptor is thought to facilitate the metastasis of mesenchymal BCSCs. A system-wide analysis of phosphorylation events identified a novel signaling pathway emanating from CXCR4 that activates protein kinase A (PKA) probably through atypical A kinase anchoring protein (AKAP). Active PKA feeds into MAP kinase-activated protein kinase 2-like (MAPKAP2) pathway which eventually stimulates the extracellular signal-regulated kinase (ERK) pathway in BCSCs (150, 152). Activation of ERK2 is critical as ERK2 is known to directly phosphorylate the TF Snail1 and induce its nuclear translocation (153). Nuclear-localized Snail1 is stable and functions as a TF which can generate and maintain BCSCs. Knockdown of CXCR4 abrogated tumor growth in mouse xenograft model (154). Moreover, in mouse mammary carcinoma model, CXCR4 was found to regulate both primary and metastatic breast cancer (155). Recently, the anti-neoplastic agent Balixafortide (a potent and selective CXCR4 antagonist) (Polyphor) in combination with Eribulin (non-taxane, anti-microtubule drug) has been successfully employed in stage IV breast cancer in a phase Ib/proof of concept clinical trial (NCT01837095). During the dose-escalation phase of the trial, the drug combination was tolerated well and no dose-limiting toxicities were observed. The objective response was observed in 30% (16 out of 54) and stable disease in an additional 46% (25 out of 54) of the stage IV patients. Based on this, balixafortide has been fast-track designated by food and drug administration (FDA) for its use in advanced, metastatic breast cancer (MBC) (156). The trial outcome suggests that balixafortide-eribulin combination chemotherapy has promising potential in heavily pretreated patients with MBC and warrants further investigation through randomized trials.

Enantiomeric RNA (L-RNA) aptamers mimicking ligands of receptors can be employed to inhibit activation of key signaling pathways. The RNA aptamer mimicking the ligand CXCL12, NOX-A12 (Olaptesed pegol), seems to control the activation of CXCR4 (157). NOX-A12 binds to two key sites in CXCL12 in order to disrupt its activity and target them for degradation. This has entered into clinical trial in patients (Noxxon Pharma-AG) (158). These will be less toxic compared to the use of small molecule inhibitors or even immunotherapy. Interestingly, combined employment of NOX-A12 and PD-1 blockade enhanced T cell and NK cell infiltration (159) and there are some ongoing clinical trials for the combination therapy involving NOX-A12 and PD-1 inhibitors in different types of cancer.

### Tyrosine Kinases

#### Human Epidermal Growth Factor Receptor 2 (HER2)

HER2 may play a role in the expansion of BCSCs in luminal cell lines and HER2^+^ breast tumors by upregulating drug transporters and the chemokine receptor CXCR4. HER2 amplification is linked to an early onset of metastasis through increases in the efficiency of mammosphere formation and expansion of the ALDH^+^ cell population (146, 148). The inhibition of HER2 decreases the invasive and tumorigenic potential of breast cancer cells (160), but HER2 modulation in BCSC could produce resistance to HER2 inhibitors such as Trastuzumab (120). In this situation, the employment of Pertuzumab which inhibits HER2 dimerization with other HER receptors may overcome the resistance. Pharmacological inhibition of HER2 using Lapatinib reversed the MDR mediated by ABCB1 and ABCG2 by directly inhibiting their transport function (142). This result suggests a possible link between ABC transporters and HER2 signaling. The mammosphere formation efficiency (MFE) was reduced regardless of HER2 status and more pronounced in BCSCs with HER2 expression by decreasing their proliferation but not self-renewal (161).

There are many ongoing clinical trials that target HER2 in combination with other approaches. HER2-sensitized dendritic cell (DC) vaccine will be employed to improve the response to breast cancer therapy and in particular preventing recurrence (NCT03630809). A phase II randomized study has started to evaluate the efficacy of anti-PD1 therapy (Pembrolizumab) with concurrent alphavirus-like replication particles containing self-amplifying replicon RNA for HER2 (VRP-HER2) vaccine in increasing the tumor infiltrating and peripheral blood immune response upon administration of the VRP-HER2 vaccine. This is for patients with advanced HER2-overexpressing breast cancer (NCT03632941). There are also numerous ongoing peptide- or domain-based anti-HER2 vaccine clinical trials (NCT02276300, NCT03793829, NCT01632332, and NCT01526473).

#### Focal Adhesion Kinase (FAK) and Rho GTPases

Bidirectional signaling operates between Rho GTPases and the focal adhesion kinase (FAK). Rho GTPases are reported to govern a variety of cellular processes including a prominent role in the regulation of cell migration. The typical Rho family members such as RhoA, Rac1, and Cdc42 function by cycling between an active GTP-bound and inactive GDP-bound conformations. They are regulated by guanine nucleotide exchange factors (GEFs), GTPase-accelerating proteins (GAPs), and GDP-dissociation inhibitors (GDIs). Among the Rho family members, Ras homolog gene family member C (RhoC) has been reported to impart tumor cell plasticity and is essential for metastasis (162–165). Functionally, RhoC co-ordinates cell motility and actomyosin contractility. RhoA and RhoC have been demonstrated to display a reciprocal relationship in TNBC cells. RhoA impedes tumor cell invasion while RhoC promotes it (166). With regard to breast cancer stemness, the expression of RhoC segregates with ALDH positivity and it impacts the frequency of CSCs found in a previous tissue microarray where 136 breast cancer tissues were analyzed (164). When RhoC was knocked down (RhoC-KD) in ALDH^+^ cells, tumor initiation was severely impaired (i.e., no induction in the RhoC-KD group vs. 5/9 tumors formed in non-silencing control when 50 CSCs are injected in each group) (164). RhoC has been suggested to work through α5-integrin and activate *Src*-FAK signaling cascade in regulating metastasis (167).

FAK plays a critical role in BCSCs and forms and attractive target. Inhibition of FAK signaling seems to selectively target BCSCs (168). Mammary epithelial-specific ablation of FAK suppresses tumorigenesis by targeting BCSCs (169). Interestingly, FAK forms a ternary complex with the cytosolic connexin26 and the transcription factor Nanog. This ternary complex is involved in the self-renewal of BCSCs of TNBC origin (170) thus forming an attractive target in BCSCs. ST8SIA1 regulates ganglioside GD2 expression in BCSCs. Interestingly, ST8SIA1 is highly expressed in primary TNBC. Genetic ablation of ST8SIA1 inhibited mammosphere formation in BCSCs. Importantly, T8SIA1-KO TNBC cells were inhibited in its tumorigenic capacity in a mouse xenograft model. Mechanistically, this process involved activation of the FAK-AKT-mTOR signaling pathway in GD2^+^-BCSCs (171). In another study, inhibition of FAK activity by VS-4718 or VS-6063 preferentially targeted BCSCs in cell lines as well as *ex vivo* cultured human primary breast cancer specimens. In a mouse xenograft TNBC model, administration of VS-4718 or VS-6063 reduced the BCSCs in the tumor significantly. The tumor-initiating ability is also reduced in the limiting dilution assay *in vivo* (172). Anti-FAK inhibitor Defactinib along with anti-PD1 therapy is in a clinical trial against solid tumors (NCT02758587).

### Serine-Threonine Kinases

#### Cyclin-Dependent Kinase 4/6

SOX2 can elevate the level of Cyclin D1 through up regulation of its transcripts through transactivation. Cyclin D1 would bind to cyclin-dependent kinase 4/6 (CDK4/6) and form the Cyclin D1-CDK4/6 complex that activates BCSC proliferation and clonogenicity. Inhibiting CDK4/6 with Palbociclib would prevent CDK4/6 activation and would thereby nullify SOX2-directed Cyclin D1. CDK4/6 inhibitors are also promising in chemoresistant cases of HER2^+^-breast cancer. Importantly, blocking the activity of CDK4/6 synergized with immune checkpoint blockade enhanced the cancer cell immunogenicity and subsequent clearance by cytotoxic T-cells (173–175). Blocking CDK4 activity reduced the stemness and efficiently eliminated chemoresistant cells (101). So CDK4/6 is an attractive target if Cyclin D1 expression is high in the tumor biopsy. A clinical trial is in place targeting CDK4/6 (SHR6390) and HER2 (Pyrotinib) in advanced breast cancer (NCT03993964). There is an ongoing clinical targeting CDK4/6 only (NCT03310879).

#### Aurora Kinase A

Aurora kinases are a family of mitotic serine/threonine protein kinases comprised of Aurora A (AURKA), Aurora B (AURKB) and Aurora C (AURKC) kinases. AURKA is involved in duplication of centrosomes and AURKB orchestrates mitotic events (176–178). The transcription factor Forkhead box subclass M1 (FOXM1) recruits nuclear AURKA to transactivate FOXM1 target genes in a kinase-independent manner in BCSCs (99). The positive feedback loop with co-operation between AURKA and FOXM1 sustains a high level of expression of both proteins. Both AURKA and FOXM1 promote maintenance and self-renewal of BCSCs (99). Additionally, the nuclear AURKA interacts with heterogeneous nuclear ribonucleoprotein K (hnRNPK) and activates the MYC promoter leading to expression of MYC. As a result, the stemness of BCSCs is enhanced (179). Aberrant AURKA activity can induce phosphorylation of SMAD5 (homolog 5 of the Drosophila protein, mothers against decapentaplegic (MAD) and the *Caenorhabditis elegans* protein Sma) that subsequently promotes the expression of CD44 leading to gain of chemoresistance (180).

There is an ongoing phase Ib trial examining Aurora A Inhibitor (Alisertib; MLN8237) in combination with a dual TORC1/2 inhibitor (MLN0128) in patients with advanced solid tumors with an expansion cohort in metastatic TNBC (NCT02719691).

#### NF-κB Inducing Kinase (NIK)

The “nuclear factor of kappa light polypeptide gene enhancer in activated B cells” (NF-κB) pathway has been implicated in transcriptional regulation of genes related to survival, proliferation, angiogenesis, metastasis, and immune responses (181). NF-κB inducing kinase (NIK) or Mitogen-activated protein kinase kinase kinase 14 (MAP3K14) is reported to enhance stem cell markers, and growth in BCSCs *in vitro* and *in vivo* (182). NIK can activate both canonical and non-canonical pathways by inducing phosphorylation and degradation of inhibitor of κB (IκB). The canonical pathway is mediated by the transcriptional activity of the p50:p65 dimer, whereas the non-canonical pathway is transcriptionally controlled by the p52:RelB dimer (183, 184). Physiologically, NIK plays an important role in the maintenance of the embryonic pluripotent stem cell state and mammary gland development. This may suggest a potential role for NIK in maintenance of BCSCs (185–188). NIK-IKKα was shown to regulate ErbB2-induced mammary tumorigenesis in a preclinical model through the nuclear export of p27/kip1 which supports the proliferation and expansion of BCSCs (189). Recently, NIK was shown to regulate the expression of genes linked to stemness through activation of ERK1/2 and the NF-κB pathways along with the correlative expression between ALDH1 and NIK in breast cancer patients tissue samples and the knockdown of NIK impaired tumorigenic potential (182).

### Epigenetic Targets

Epigenetic modifications play a key role in self-renewal, heterogeneity (190) and plasticity of BCSCs (191). Adaptive chromatin remodeling (methylation/demethylation of gene promoters and different lysine residues in histones) may result in differential regulation of proteins leading to chemoresistance and plasticity. An upregulation of drug transporters would lead to chemoresistance and increased viability following therapy. Modulation of transcription factor (TF) networks have been observed in BCSCs. Poised chromatin at “Zinc Finger E-Box Binding Homeobox 1” (ZEB1) sites was reported to play a role in generating CSCs in response to ligands in the TME (192). Snail1 up regulates expansion and activity of BCSCs through repression of p53 (71). The pluripotency factor SOX2 has been implicated in up regulating the activity of the multidrug transporter ABCG2 and the TF Twist1 (112). The level of SOX2 also correlated with the tumor size and expression of epidermal growth factor receptor (EGFR) and cyclin-dependent kinase 5/6 (CDK5/6). Histone deacetylases (HDAC), HDAC1 and HDAC7, are selectively amplified in BCSCs and so these can be targeted (193) either individually or by combination therapy (DNMT and HDAC inhibitors). Histone lysine-specific demethylase 1 (LSD1 or KDM1) is involved in stemness and can serve as a potential target in BCSCs (114). The key clinical advantage is that the epigenetic states are reversible and this vulnerability should be clinically targeted.

The Anti-HDAC6 inhibitor (KA2507) is being examined clinically in patients with PD-L1 expressing solid tumors which have relapsed or are refractory to prior treatment (NCT03008018). A phase I trial is in place targeting LSD1 with the inhibitor (Seclidemstat) in patients with advanced solid tumors (NCT03895684).

#### Quiescent BCSCs (qBCSCs)

Quiescent CSCs play important roles in tumor dormancy, relapse and resistance to therapy. SET domain-containing protein 4 (SETD4) was demonstrated to be important for the maintenance of qBCSCs. SETD4 trimethylates the side chain of 2nd lysine residue of histone H4. This creates the formation of H4K20me3 (heterochromatin) on the promoter regions leading to silencing of genes that regulate qBCSCs. SETD4-generated qBCSCs were resistant to therapy and promoted tumor relapse in a mouse model and correlated with malignancy and chemoresistance in patients. Importantly, qBCSC underwent asymmetric division into a small quiescent BCSC and a bigger and active daughter cell that proliferates and generates normal tumor cells. Single-cell sequence analysis indicated that SETD4^+^-qBCSCs cluster together among the heterogeneous BCSCs (26).

#### Non-coding RNAs

##### MicroRNAs and long non-coding RNAs

Micro RNAs (miRs) and long non-coding RNAs (lncRNAs) play a key role in the sustenance and also the heterogeneity of BCSCs in TNBC (194). miR-600 acts as a bimodal switch and pushes BCSCs into differentiation and *vice versa* when miR level was regulated (195). miR-519d overcomes cisplatin-resistance in BCSCs by downregulating the expression of MCL1 making them less viable. miR-199a directly repressed nuclear receptor corepressor (NCOR) and this protected BCSCs from interferon-based induction of senescence and differentiation (196). miR-100 inhibits self-renewal of BCSCs and tumorigenesis (197). LncRNA H19 is responsible for glycolysis and maintenance of BCSCs (198, 199). LncRNA HOTAIR is upregulated in BCSCs derived from MCF7 and MDA-MB-231 cells. HOTAIR transcriptionally downregulates miR-34a level which spares degradation of SOX2 mRNA and in turn increased SOX2 protein levels contributing BC stemness (200). Similar to HOTAIR, the lnc RNA “metastasis-associated lung adenocarcinoma transcript-1” (MALAT-1) plays a critical role in maintaining the BC stemness. First, the level of MALAT-1 was higher in BCSCs than the parental MCF7 cells. Silencing of MALAT-1 led to reduction in the number of BCSCs and the mammosphere formation efficiency. Furthermore, there was reduced proliferation, colony formation, migration and invasion of BCSCs *in vitro* (201). Targeting of lnc RNA NRAD1 produced cells with less BCSC characteristics (202). Interestingly, mesenchymal stem/stromal cells trigger a lncRNA LINC01133 pathway in neighboring TNBC cells which upregulates pluripotency factor “Kruppel-Like Factor 4” (KLF4). This pushes tumor cells into cancer stemness (203). Also, lnc RNA FEZF1-AS1 has been shown to promote BC stemness and tumorigenesis via targeting miR-30a/Nanog axis (204). Long non-coding RNA in the aldehyde dehydrogenase 1 A pathway (NRAD1) has been suggested to be a potential target in TNBC and BCSCs. Targeting of NRAD1 using the ASO approach resulted in reduced cell survival, tumor growth, and the number of cells with CSC characteristics (202).

### “Metabostemness”

CSCs employ either glycolysis or mitochondrial oxidative phosphorylation (OXPHOS) depending on the temporality and the microenvironment or the niche in which they are placed. In the quiescent mode, BCSCs utilize glycolytic pathway for their energy needs. In the proliferative state, BCSCs employ OXPHOS mode of energy derivatization (18, 74). So targeting the metabolic flexibility of BCSCs between OXPHOS and glycolysis may force them into a unilateral OXPHOS or glycolytic mode and may sensitize them to anti-CSC inhibitors. A two “metabolic hit” strategy has been proposed for the eradication of CSCs. Doxycycline has been shown to impair the mitochondrial respiration and a second hit targeting the glycolysis will be effective in elimination of CSCs (205).

#### “Energetic” Breast Cancer Stem Cells (e-BCSCs)

Based on the energetic profile, a new subset of hyper-metabolic, proliferative BCSCs (called e-BCSCs) driven by mitochondrial energy has been identified. This reflects the presence of metabolic heterogeneity in BCSCs. These eBCSCs are more glycolytic with elevated oxidative metabolism and increased mitochondrial mass. These were ALDH^+^ with enhanced anchorage-independent growth and NRF2-mediated anti-oxidant response signature. The e-BCSCs can be effectively targeted by OXPHOS and CDK4/6 inhibitors. Therefore, mitochondrial inhibitors to target this subset of highly active BCSCs should be developed (25, 206).

A small molecule inhibitor against mitochondrial electron transport chain complex I (IACS-010759) has been demonstrated to inhibit cell growth in 13 of the 16 TNBC cell lines employed. An ongoing clinical trial (NCT03291938) is in place with IACS-010759 in advanced breast cancer patients (207). Another similar drug (ME-344) against mitochondrial complex I is in phase I clinical trial in breast cancer patients (NCT02806817).

### Redox Pathways

#### NF-E2-Related Factor 2 (NRF2)

Newer data has identified NF-E2-related factor 2 (NRF2) transcription factor as a novel biomarker for BCSCs. One study has shown that NRF2 expression increases in drug resistant BCSCs (208). NRF2 is a master regulator of cell redox homeostasis. It performs its regulatory function by up regulating genes that have an antioxidant response element (ARE). The work of Wu et al. (208) revealed that NRF2 conferred resistance to multiple drugs in BCSCs by keeping ROS level reduced during the drug treatment. In a recent discovery, CD44^+^-BCSCs showed co-localization of NRF2 with CD44, and found that NRF2 expression was dictated by CD44-p62 signaling (209). Importantly, co-inhibition of NRF2 or downstream thioredoxin and glutathione antioxidant pathways and glycolysis has been shown tom induce terminal differentiation of both mesenchymal and epithelial BCSCs and induction of apoptosis (74). Furthermore, this inhibition suppressed tumor growth, tumor-initiating potential and importantly metastasis by eliminating both mesenchymal and epithelial BCSCs (74). Additionally, NRF2 has been implicated in other CSC types including ovarian (210) and acute myeloid leukemia (AML) (211).

### Miscellaneous

#### Sirtuin1 (SIRT1)

Sirtuin1 (SIRT1) is a nicotinamide adenine dinucleotide (NAD)-dependent deacetylase involved in both cellular stress and longevity. The hallmark function of SIRT1 is to enhance cell survival through the deacetylation and inactivation of p53 (212). An increase in SIRT1 expression levels in drug resistant cancer cell lines induce deacetylation and activation of FOXO1 which upregulates drug transporters MDR1 (213) and MRP2 (214). SIRT1 is a key facilitator in stem cell biology as well. For instance, mouse embryonic stem cells which lack SIRT1 have a delayed capacity to differentiate through the ability of SIRT1 to repress the expression of Dnmt3l (215). In addition, SIRT1 inhibits p53-mediated suppression of Nanog, a pluripotency transcription factor involved in the maintenance of BCSCs (216). SIRT1 is upregulated in CD44^+^/CD24^−^ mesenchymal BCSCs (217). In another report, SIRT1 is downregulated in ALDH1^+^ epithelial BCSCs and is reported to stabilize the EMT inducer PRRX1 and indirectly inhibits the stemness factor KLF4 (218). Future studies will need to elucidate the role of SIRT1 in mesenchymal and epithelial BCSCs and clear out the controversies involved. Alternatively, relative abundance of other SIRT isoforms may contribute to differential outcome observed. A mechanistic understanding of the role of SIRT1 in each type of BCSCs will justify future therapeutic intervention as both small molecule activators and inhibitors are commercially available for SIRT1 (219). Lastly, it is also interesting to note that c-MYC has been reported to activate SIRT1 which in turn promotes c-MYC function (220).

#### Targeting Other Signaling and Survival Pathways in BCSCs

The MCL1 inhibitor S63845 has been reported to have success against BCSCs arising out of HER2+ and TNBC (221). Targeting the Wnt/β-catenin signaling pathway showed promising results in reducing the metastatic potential by altering BCSC activity in a preclinical mouse model (222). Moreover, loss of the tumor suppressor Liver-kinase B1 (LKB1) led to an increase in the number of BCSCs (223) and elevated expression of OCT4, Nanog, and SOX2. Interestingly, a plant bioactive molecule called “Honokiol” effectively upregulated LKB1 protein levels that abrogated the stem phenotype (224). In addition, co-targeting of Notch ligand production and IL-6 receptor in human breast cancer cell lines and PDX xenografts was beneficial in reducing the number of BCSCs (225). A caveat would be targeting the Notch pathway may be detrimental to the immune system (226). Finally, Insulin-like growth factor-2 (IGF-2) induced NF-κB activity and blockade of the IGF-2 signaling reduced tumorigenesis in a PDX model enriched with BCSCs (227).

#### Tinkering With Lysosomes

Drug screening identified salinomycin as a selective agent against BCSCs by sequestering iron in lysosomes that led to ferroptosis of CSCs (228, 229). In particular, C20-O-acylated analogs of salinomycin performed better in terms of efficacy (230). Ferroptotic agents have been shown to selectively kill BCSCs (231). The anti-malarial drug chloroquine (CQ) was reported as a sensitizing agent to paclitaxel through inhibition of autophagy in TNBC cells. This reduced the number of CD44^hi^/CD24^−/low^-BCSCs in both preclinical and clinical settings (232). Mechanistically, CQ worked to inhibit the Janus kinase 2 (Jak2)-signal transducer and activator of transcription 3 (JAK2-STAT3) signaling pathway. (233). The downside of CQ is that it favors the accumulation of CD3^+^/CD4^+^/FOXP3^+^ regulatory T cells (T_regs_) (234).

## Concluding Remarks

In this review, we have explored the molecular origin of BC stemness and chemoresistance and have identified several emerging molecular targets that are vital for BCSCs. These targets could be employed to overcome chemoresistance mediated by BCSCs. By simultaneous targeting both BCSCs and bulk tumor cell populations, the problem arising out of interconversion between bulk tumor and stemness state could be contained. This will prevent tumor relapse and increase patient longevity. Additionally, metabolic vulnerabilities should be combined with novel pharmacological targets. Overall, combinatorial therapy involving emergent vulnerable nodes in receptor and redox signaling pathways, survival, self-renewal, drug efflux transporters, and metabolism would pave the way for effective modalities of therapy and attain favorable prognosis in the metastatic breast cancer.

## Author Contributions

SS: contributed to introduction, Table 1, NIK section, and oversaw several sections. CH: contributed a section on SIRT1 and drew Figure 2. AMCT: composed Table 2 and a section on NRF2. BS: contributed a section on AurK. AKT: contributed a section on efflux transporters and edited the whole manuscript. RR: edited the manuscript and contributed intellectual concepts. DR: contributed to all sections, wrote all other sections not mentioned above, edited and intellectually co-ordinated the complete manuscript and drew Figure 1.

### Conflict of Interest

The authors declare that the research was conducted in the absence of any commercial or financial relationships that could be construed as a potential conflict of interest.
